# The Myelin Sheath as a Multilamellar Electromechanical System: Bilayer Stack Analogs, Energy Buffering, and Biological Memory

**DOI:** 10.3390/membranes16090285

**Published:** 2026-08-28

**Authors:** Dima Bolmatov, Zack Woodel, Igor M. Gussev, Miguel Turrero García, Erik B. Watkins, Yong Q. Cai, Ilia N. Ivanov

**Affiliations:** 1 Department of Physics and Astronomy, Texas Tech University, Lubbock, TX 79409, USA; 2 Department of Cell Physiology and Molecular Biophysics, TTUHSC School of Medicine, Lubbock, TX 79430, USA; 3 Department of Electrical and Computer Engineering, Texas Tech University, Lubbock, TX 79409, USA; 4 Neutron Scattering Division, Oak Ridge National Laboratory, Oak Ridge, TN 37831, USA; 5 Department of Biological Sciences, Texas Tech University, Lubbock, TX 79409, USA; 6 National Synchrotron Light Source II, Brookhaven National Laboratory, Upton, NY 11973, USA; 7 Center for Nanophase Materials Sciences, Oak Ridge National Laboratory, Oak Ridge, TN 37831, USA

**Keywords:** myelin sheath, multilamellar lipid stacks, electromechanical coupling, phonon-mediated energy buffering, biological memory

## Abstract

The myelin sheath has traditionally been viewed as a passive electrical insulator that accelerates nerve impulse propagation. Recent experimental studies, however, indicate that myelin is a dynamic biological material whose structure and hydration state adapt to neuronal activity, metabolic conditions, and environmental perturbations. Building on these observations, we propose a conceptual framework that treats myelin as an adaptive electromechanical multilamellar interface, in which coupled lipid bilayers, hydration layers, and interlayer interactions influence energy dissipation, structural adaptation, and history-dependent behavior. Within this framework, collective excitations and delayed relaxation processes are hypothesized to contribute to transient energy storage and adaptive responses to electrical activity in the integrated axon–glia system. We further argue that testing this framework requires multimodal characterization combining electrophysiology with neutron and X-ray scattering, terahertz spectroscopy, and data-driven analysis to establish quantitative relationships between membrane structure, dynamics, and function. This work outlines experimentally testable predictions and provides a foundation for investigating how electromechanical adaptation of myelin may contribute to normal neural function and the early biophysical changes associated with demyelinating disease.

## 1. Introduction

The myelin sheath is recognized as one of the most lipid-rich and structurally specialized biological materials found in nature [1]. It consists of tens to hundreds of tightly packed lipid bilayers, which are wrapped around neuronal axons. This unique composition allows myelin to facilitate rapid and energy-efficient signal propagation along neurons. In addition to its role in electrical conduction, the myelin sheath acts as a dynamic interface that connects electrical activity, metabolism, and cellular homeostasis within the nervous system [2]. In the central nervous system (CNS), myelin is produced by oligodendrocytes (OLs), highly specialized glial cells capable of myelinating up to approximately sixty distinct axonal segments. The geometry of the myelin sheath, including its thickness and internode length, is dynamically tuned to axonal diameter and neuronal activity, reflecting the remarkable ability of oligodendrocytes to sense and respond to their local environment [3]. During development, oligodendrocytes generate multiple short sheaths that are selectively retracted, stabilized, or expanded in a Ca^2+^-dependent manner as mature myelination patterns emerge [4]. The formation and maintenance of myelin additionally depend on metabolic support from neighboring glial cells, particularly astrocytes, highlighting the tightly coupled structural, electrical, and metabolic ecosystem that underlies myelin function.

Several characteristics make myelin uniquely suited for investigating adaptive membrane behavior. First, myelin contains approximately 70–80% lipids by dry weight, making it one of the most lipid-dense structures in the body [5,6]. Second, its multilamellar architecture creates a highly ordered stack of coupled membranes that spans multiple length scales, from molecular organization within individual bilayers to micron-scale sheath geometry. Third, myelin continuously remodels throughout life in response to neuronal activity, learning, aging, injury, and disease Together, these properties position myelin as an exceptional biological platform for exploring the interplay between structure, dynamics, energy flow, and memory. Its periodic multilamellar organization, confined hydration layers, high lipid content, and MBP/PLP-mediated stabilization create a tightly coupled membrane system whose structural and electromechanical properties are continuously influenced by axon–glia interactions. These well-established features provide the physical foundation for the conceptual framework proposed in this work, in which the coupling between membrane architecture, hydration, and electrical activity gives rise to adaptive and history-dependent behavior.

Although myelin has historically been regarded primarily as a passive electrical insulator that increases membrane resistance and enables saltatory conduction by clustering voltage-gated sodium channels at the nodes of Ranvier [7], contemporary research has established that it is also a metabolically active, structurally dynamic tissue that participates in activity-dependent myelination, oligodendrocyte signaling, and adaptive remodeling [8]. Building upon these biological advances, we propose a complementary physica–material framework that links these processes to membrane hydration, collective dynamics, and electromechanical responses. Within this framework, myelin is viewed not only as an electrical insulator but also as a dynamic electromechanical system whose structural organization governs its physical response to electrical activity. While the traditional description successfully explains many aspects of nerve conduction, it does not fully capture the increasingly dynamic picture emerging from modern neuroscience and membrane biophysics. Recent studies have shown that the early stages of myelin damage are often characterized by pronounced swelling that can precede irreversible degeneration and may, under appropriate conditions, be partially reversed through structural remodeling [9]. Clinically, alterations in myelin integrity and thickness are associated not only with demyelinating diseases such as multiple sclerosis but also with age-related cognitive decline and stress-related neurological disorders. Neuronal activity directly influences these processes: elevated activity can exacerbate myelin instability and swelling, whereas reduced activity can mitigate damage, revealing a strong coupling between electrical signaling and myelin structural integrity [9]. These observations motivate the physical–material perspective developed in this work, in which membrane architecture, hydration, and collective dynamics contribute to the electromechanical behavior of myelin.

At the molecular level, the myelin sheath is enriched in cholesterol, glycosphingolipids, and specialized proteins such as myelin basic protein (MBP) and proteolipid protein (PLP). Together, these components determine membrane elasticity, hydration, dielectric response, and phase behavior [10]. Importantly, myelin is not simply a stack of lipid bilayers but a highly organized assembly in which membrane headgroup dipoles, hydration layers, ions, and confined aqueous environments interact cooperatively. These interactions continuously reshape electrostatic screening, viscoelastic properties, and electromechanical response [11]. Consequently, myelin should be viewed not only as a structural component of the nervous system but also as a responsive material whose physical state may directly influence signal transmission, structural maintenance, and repair.

Recent advances in membrane biophysics further challenge the long-standing view of biological membranes as passive barriers [12,13,14,15]. Model membrane systems, including lipid bilayers, exhibit electromechanical coupling, nonlinear dielectric response, capacitive hysteresis, and history-dependent behavior under external stimulation [16,17]. In multilamellar lipid systems, these effects are further amplified by inter-bilayer coupling, hydration-mediated interactions, and structural periodicity, giving rise to collective non-equilibrium dynamics [18]. Recent studies have demonstrated that phospholipid membranes can exhibit persistent electromechanically induced states, remanent polarization, and long-lived collective dipolar correlations following external stimulation [19,20]. These coupled responses reshape local charge distributions and collective vibrational states, establishing a feedback loop in which electric fields reorganize membrane structure, and membrane structure, in turn, modifies the electrical response. Although these phenomena have been demonstrated primarily in model membrane systems, they motivate the hypothesis that analogous electromechanical mechanisms may also operate within the highly ordered multilamellar architecture of myelin. Direct experimental evidence for such behavior in native myelin, however, remains limited. Such observations suggest that membrane organization may possess an intrinsic capacity to transiently store information through structural and dynamical states, motivating the broader hypothesis that adaptive and memory-like behavior may also emerge in myelin [12,19].

Hydration plays a crucial role in these processes, as interfacial water forms a structured and dynamic medium that governs membrane mobility, viscoelasticity, dielectric response, and phase stability [21]. Small variations in hydration, on the order of a few water molecules per lipid, can induce phase transitions, alter inter-bilayer interactions, and introduce slow relaxation processes that coexist with fast electrical responses [22]. The resulting hierarchy of timescales naturally gives rise to non-Markovian dynamics, delayed relaxation, and emergent memory-like behavior. In myelin, hydration therefore acts not merely as a passive environmental variable but as an active regulator of energy flow, structural adaptation, and functional response. In native compact myelin, these hydration-mediated effects are expected to be most pronounced at the cytoplasmic and extracellular appositions and during structural remodeling or pathological swelling, where local water content and interlamellar spacing become dynamic. By contrast, simplified model multilamellar lipid stacks provide experimentally accessible systems in which hydration can be systematically varied to isolate and quantify its contribution to membrane electromechanical behavior.

Importantly, myelin does not experience the full transmembrane action-potential waveform [2]. Instead, electrical activity is filtered through the axon–myelin system, producing slowly varying local electric fields that couple to membrane dipoles, hydration layers, ions, and collective modes. Order-of-magnitude estimates indicate that these fields evolve on microsecond-to-millisecond timescales and generate local electric fields of approximately $10^{4}$ – $10^{6}\;V\;m^{-1}$ , depending on the local geometry and dielectric environment. Although substantially attenuated relative to the transmembrane electric field across the axonal membrane, these fields act over extended periods throughout the multilamellar sheath, where they may influence dipolar orientation, hydration structure, and interlamellar interactions without directly depolarizing the membrane. While the electrostatic energy associated with an individual molecular interaction is expected to be small relative to thermal fluctuations ( $k_{B}T$ ), repeated neuronal activity and cooperative interactions among coupled lipids, hydration layers, and proteins may give rise to cumulative electromechanical effects. This perspective naturally positions myelin as an active participant in information processing rather than a passive bystander to neuronal signaling.

Despite these advances, a critical gap remains: structure, dynamics, and function are typically investigated independently. Scattering techniques such as neutron and X-ray reflectometry and small-angle scattering resolve membrane thickness, periodicity, and lateral organization. Spectroscopic methods probe collective excitations, including acoustic and optical phonons that govern energy transport and storage. Functional measurements capture nonlinear and history-dependent responses. However, a unifying framework that connects these domains across scales is lacking.

Here, we propose a function-first multimodal framework for understanding biological membranes. In this framework, functional responses serve as the primary observables, while structural and dynamical measurements provide mechanistic constraints on the underlying state of the system. By integrating electrophysiology, scattering, spectroscopy, and computational analysis, it becomes possible to establish quantitative links between membrane organization, collective dynamics, energy flow, and biological function.

Within the proposed framework, membrane dynamics arise from the interplay of multiple energy sources rather than from thermal energy alone. Equilibrium thermal fluctuations provide the background that enables continuous exploration of the membrane energy landscape, while externally applied electrical fields and mechanical deformations perform work on membrane dipoles, hydration layers, and collective modes, driving the system away from equilibrium. In living systems, these physical processes may be further modulated by ATP-dependent cellular processes involved in myelin maintenance and remodeling. Together, thermal fluctuations, externally driven electrical and mechanical work, and metabolic regulation govern structural adaptation, relaxation dynamics, and the emergence of history-dependent electromechanical states.

We further argue that these processes are fundamentally heat-driven, with thermal energy acting as a common energetic currency linking electrical, mechanical, and structural transformations [12,19]. The interplay between hydration, electromechanical coupling, and collective dynamics gives rise to adaptive, history-dependent behavior that is inherently non-equilibrium and non-ergodic. Integrating functional measurements with multimodal structural and dynamical characterization enables the identification of emergent principles governing biological membranes and their role in adaptation, memory, and disease.

This Perspective reframes the myelin sheath not as a passive insulator, but as an adaptive electromechanical multilamellar interface capable of energy buffering, structural remodeling, and memory. Understanding how composition, hydration, electrical activity, and collective dynamics regulate this behavior opens new directions for exploring development, aging, and disease, including the possibility of detecting the earliest stages of demyelination at the biophysical level and providing opportunities for intervention before irreversible structural damage occurs.

## 2. Function as the Primary Observable

Traditional approaches to studying biological membranes have largely emphasized structure and composition as primary descriptors of function. While these approaches have yielded critical insights, they often treat function as a downstream consequence rather than a measurable, dynamic quantity. In contrast, we propose that function itself should serve as the primary observable. Here, “function” is defined operationally as the measurable response of a membrane system to external electrical or mechanical stimulation. Rather than representing a single quantity, it is characterized through complementary observables, including capacitance and conductance dynamics, lamellar spacing changes, swelling and recovery kinetics, viscoelastic dissipation, and spectroscopic signatures of collective modes. Together, these observables provide direct access to the underlying physical processes governing membrane behavior.

In this framework, functional measurements are not merely endpoints but high-dimensional signals that encode the coupled electromechanical, structural, and dynamical state of the system. Techniques such as electrophysiology, quartz crystal microbalance with dissipation (QCM-D), and broadband spectroscopies provide complementary access to different aspects of membrane function across multiple timescales and length scales. Together, these techniques probe electrical response, hydration-mediated adaptation, viscoelastic relaxation, and collective dynamical behavior, enabling the direct observation of nonlinear, history-dependent, and non-equilibrium processes that emerge from the coupling between structure, dynamics, and function.

Electrophysiological measurements, including patch-clamp techniques, provide direct access to ionic transport, membrane conductance, capacitance, and voltage-dependent responses in native myelin-associated cells, axon–myelin preparations, and simplified model membrane systems [23,24]. In this work, electrophysiological measurements are discussed in the context of complementary experimental platforms. Native oligodendrocytes and axon–myelin preparations provide the physiological setting for studying myelin function, whereas electrically stimulated model lipid bilayers and multilamellar membrane stacks enable controlled investigation of electromechanical coupling and memory-like behavior under well-defined experimental conditions. While oligodendrocytes are not classically electrically excitable cells, electrophysiological measurements can nevertheless reveal subtle changes in membrane conductance, capacitance, ion homeostasis, and electromechanical state that accompany structural adaptation and myelin remodeling. Such measurements therefore provide a functional window into membrane organization and its response to external perturbations. Beyond their traditional role in characterizing ion channel activity, these measurements can reveal deviations from linear capacitive behavior, including hysteresis in current–voltage and charge–voltage relationships. In lipid bilayers and living systems alike, these responses reflect the coupling between electrical stimulation, membrane deformation, dipolar alignment, and ion redistribution, providing insight into how biological membranes respond to and retain information about prior stimuli.

Importantly, these responses span a broad hierarchy of temporal scales. Rapid processes, occurring on microsecond-to-millisecond timescales, include ion transport, membrane charging, and electrical responses associated with neuronal activity. Intermediate timescales involve membrane deformation, dipolar reorganization, hydration-layer restructuring, and collective relaxation processes. At longer timescales, extending from minutes to days, changes in lipid composition, protein organization, metabolic state, and gene expression can further reshape membrane properties. The coexistence of these coupled processes across multiple temporal scales provides a natural foundation for memory, adaptation, and history-dependent behavior.

Complementary to electrophysiology, QCM-D provides a sensitive probe of mass uptake, viscoelasticity, and interfacial dynamics [25]. In multilamellar membrane systems, QCM-D measurements capture hydration-driven changes in membrane thickness, rigidity, and dissipation, revealing slow relaxation processes that are not accessible through purely electrical measurements. The coexistence of fast electrical responses and slow mechanical relaxation introduces intrinsic timescale separation, a hallmark of non-Markovian systems. Hysteresis observed in sorption–desorption cycles and viscoelastic response suggests the presence of history-dependent hydration-mediated structural states. Experimentally, hydration-mediated memory may be tested by subjecting myelin-like multilamellar membrane systems to repeated hydration–dehydration cycles, electrical stimulation followed by relaxation, or controlled mechanical perturbation while continuously monitoring QCM-D frequency and dissipation. The proposed mechanism predicts path-dependent hysteresis between loading and recovery trajectories, incomplete recovery to the initial baseline, or relaxation times that depend on the magnitude and history of prior perturbations. Such signatures would distinguish history-dependent structural adaptation from a fully reversible equilibrium hydration response.

Broadband spectroscopic techniques, including terahertz time-domain spectroscopy (THz-TDS), extend this functional perspective into the frequency domain by probing collective dipolar and vibrational dynamics. These measurements provide access to low-energy excitations associated with hydration layers, lipid headgroup motions, and collective modes coupled to both electrical and mechanical degrees of freedom. As such, THz-TDS serves as a bridge between microscopic dynamics and macroscopic functional response, connecting molecular-scale fluctuations to emergent membrane behavior [26].

A key feature of these functional measurements is their inherently high dimensionality. Each experiment generates rich datasets spanning voltage, frequency, time, and environmental parameters such as temperature, hydration, and ionic strength. Rather than reducing these data to simplified scalar quantities, we emphasize their role as informational fingerprints of the system’s state. This perspective naturally aligns with data-driven approaches, where automated experimentation and computational analysis enable the exploration of complex parameter spaces and the identification of hidden correlations.

Within such a framework, electrophysiology, QCM-D, THz-TDS, and X-ray or neutron scattering measurements are performed under common electrical stimulation and hydration protocols. Electrophysiology provides conductance, capacitance, charge-transfer, and hysteresis dynamics; QCM-D quantifies hydration-dependent viscoelastic relaxation; THz-TDS probes collective dipolar and vibrational dynamics; and scattering measurements determine lamellar spacing, membrane thickness, and structural organization. These complementary datasets are integrated to infer latent variables representing the underlying electromechanical state, hydration state, structural order, and energy landscape of the membrane system. The inferred model then guides subsequent closed-loop experiments by identifying new stimulation or hydration protocols predicted to produce specific adaptive responses. Model predictions are validated experimentally by testing whether the inferred latent variables accurately predict independent observables, including hysteresis evolution, relaxation kinetics, phonon spectra, or structural remodeling under previously unseen experimental conditions.

Importantly, this function-first approach is particularly well suited for studying non-equilibrium systems such as biological membranes. The presence of hysteresis, slow relaxation, and path dependence indicates that these systems cannot be fully described by equilibrium thermodynamics alone. Instead, their behavior reflects a complex interplay between external driving, internal degrees of freedom, and dissipative processes. Within the proposed framework, memory-like behavior is distinguished from slow equilibration by its dependence on stimulus history rather than on elapsed time alone. A purely viscoelastic or hydration-driven relaxation process is expected to converge to the same equilibrium state regardless of the sequence of prior perturbations. By contrast, memory-like behavior predicts that the system response depends on the magnitude, duration, or sequence of previous electrical, mechanical, or hydration stimuli. Experimentally, such behavior would be evidenced by persistent hysteresis, path-dependent relaxation trajectories, remanent changes in electrical or structural observables following stimulus removal, or by reproducible differences in system response despite identical final experimental conditions but different stimulation histories.

We therefore posit that biological membranes, and in particular multilamellar systems such as myelin, should be understood as adaptive, memory-bearing materials whose function emerges from coupled electromechanical and hydration-mediated processes. Functional measurements provide the most direct window into this behavior, enabling the identification of mechanisms that are otherwise obscured when structure or dynamics are considered in isolation.

In this context, the experimental platforms developed at Texas Tech University, in conjunction with multimodal characterization at national user facilities such as Oak Ridge National Lab and Brookhaven National Lab, establish a foundation for a unified, data-driven approach to membrane biophysics. By placing function at the center of investigation, this framework enables a systematic exploration of how electrical stimulation, hydration, and structural organization give rise to emergent phenomena such as energy buffering, hysteresis, memory, and adaptive response of the membrane.

## 3. Structure: Spatial Organization and Multilamellar Architecture

Structure provides the spatial foundation upon which membrane function and dynamics emerge. In biological systems, and particularly in the myelin sheath, this structure is inherently hierarchical, spanning molecular (∼0.1–10 nm), nanoscopic (∼10–1000 nm), and mesoscopic (∼1–100 μm) length scales. Myelin is composed of tens to hundreds of stacked lipid bilayers arranged in a highly ordered multilamellar architecture, where interlayer spacing, membrane thickness, and lateral organization collectively define its physical properties [27,28].

At the nanoscale, each lipid bilayer can be characterized by its membrane thickness, area per lipid, acyl chain order, and compositional heterogeneity [22]. These structural parameters are not static; rather, they evolve in response to environmental conditions such as hydration, ionic strength, and external fields [29]. In multilamellar systems, inter-bilayer coupling introduces additional degrees of freedom, including fluctuations in interlamellar spacing, correlated undulations, and collective structural rearrangements. These effects are further modulated by cholesterol-rich membrane domains and specific membrane proteins, such as myelin basic protein (MBP), which mediate adhesion and stabilization of adjacent bilayers, effectively “zippering” the structure together [27].

Within the proposed electromechanical framework, the hierarchical architecture of myelin provides the structural basis for its adaptive electromechanical response. Membrane packing parameters—including area per lipid, acyl chain order, membrane thickness, and lipid composition—govern membrane capacitance, dielectric response, mechanical stiffness, and compressibility. Interlamellar spacing and hydration layers regulate electrostatic screening, viscoelastic relaxation, and electromechanical coupling between adjacent bilayers. MBP-mediated adhesion stabilizes the compact multilamellar architecture while facilitating mechanical coupling and energy redistribution across the sheath. Cholesterol-rich domains further modulate lateral membrane organization, collective molecular dynamics, and membrane compressibility. Together, these structural features determine how electrical energy is stored, redistributed, and dissipated throughout the multilamellar membrane, thereby shaping the adaptive electromechanical behavior proposed in this work.

**Figure 1:**
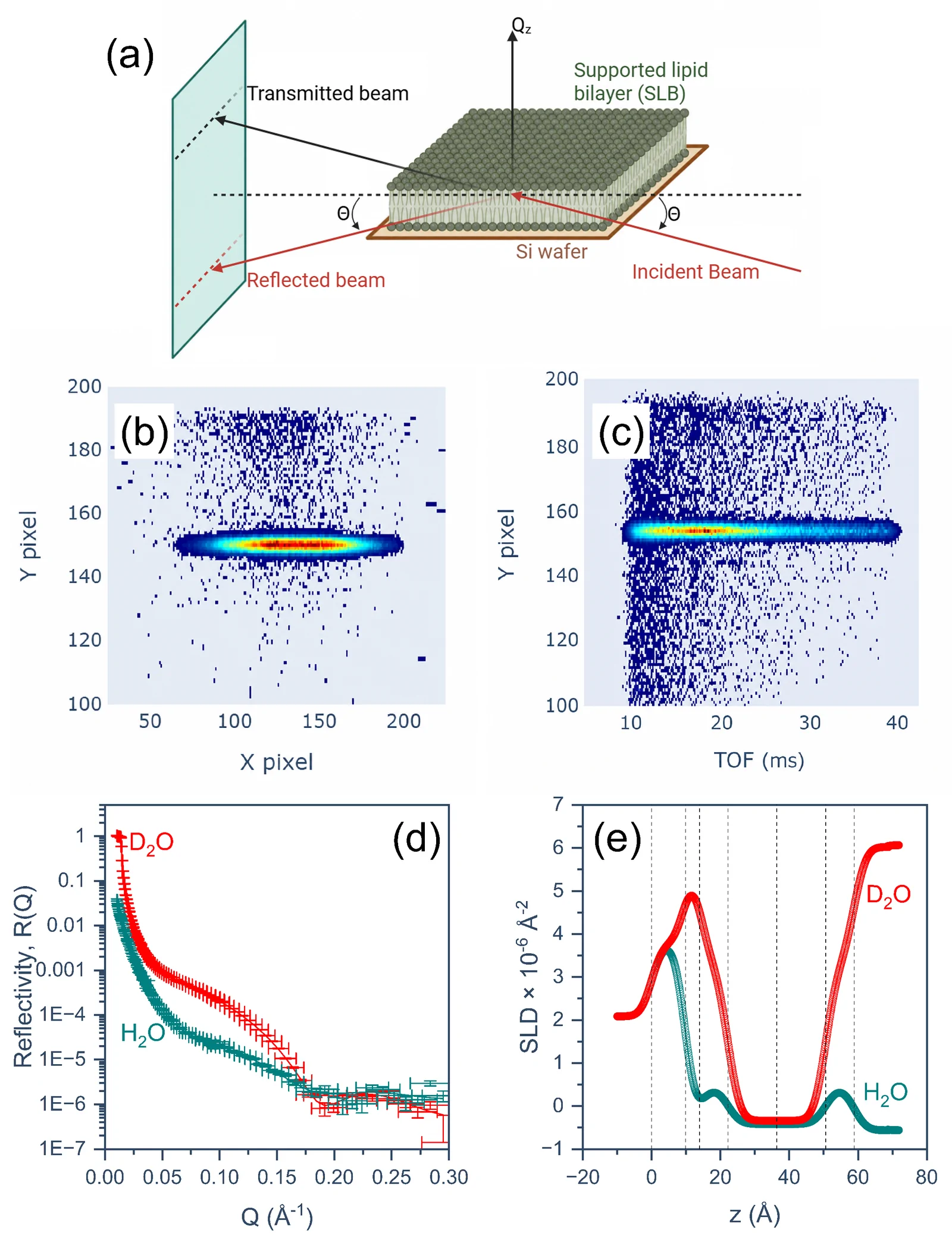
Neutron reflectometry as a structural probe of supported lipid bilayers. (**a**) Schematic representation of a neutron reflectometry experiment showing the incident, reflected, and transmitted neutron beams interacting with a supported DPhPC lipid bilayer deposited on a silicon substrate. (**b**) Representative detector image of the reflected neutron beam. (**c**) Corresponding beam profile represented in time-of-flight (TOF) coordinates. (**d**) Representative original neutron reflectometry data (symbols with error bars) and best-fit model calculations (solid lines) obtained for the supported DPhPC lipid bilayer in H_2_O and D_2_O environments. (**e**) Corresponding scattering length density (SLD) profiles reconstructed from the reflectivity analysis. The dashed vertical lines indicate the positions of the structural layers used in the slab model. Neutron reflectometry provides quantitative structural information on membrane thickness, hydration, interfacial roughness, and bilayer organization, forming the structural framework for understanding electromechanical coupling and adaptive behavior in multilamellar membrane systems such as myelin.

Neutron and X-ray scattering techniques provide complementary approaches for resolving the structural complexity of biological membranes [30]. Neutron reflectometry (NR) enables precise determination of membrane thickness, interfacial roughness, hydration profiles, and electric field-induced structural changes in supported lipid bilayers, providing sub-nanometer resolution of the out-of-plane membrane structure [31,32]. Figure 1 presents representative original neutron reflectometry measurements acquired at the Liquids Reflectometer (LIQREF) instrument at the Spallation Neutron Source, together with the corresponding fitted scattering length density (SLD) profile for a supported DPhPC lipid bilayer. The bilayer was prepared from a 2 mg/mL DPhPC solution in isopropanol using the solvent-assisted lipid bilayer (SALB) method [33]. Reflectivity profiles measured in two solvent contrasts (H_2_O and D_2_O) were fitted simultaneously using the REFL1D software package [34] to improve model stability and constrain the fitted structural parameters (Figure 1c). The resulting SLD profile (Figure 1d) provides quantitative information on supported lipid bilayer coverage, membrane thickness, headgroup and hydrocarbon chain distributions, interfacial roughness, and headgroup hydration as a function of distance from the silicon substrate (*z*).

Small-angle neutron and X-ray scattering (SANS/SAXS) extend this capability by probing lateral membrane organization, nanoscale heterogeneity, and domain formation. Together, these complementary techniques provide direct access to the hierarchical organization of multilamellar membrane systems across molecular, nanoscopic, and mesoscopic length scales [35].

A key concept that emerges from this structural perspective is that of the membrane as a spatially discretized system, where local regions, or “pixels”, of the bilayer exhibit distinct physical properties. Within this work, a *pixel* denotes a conceptual local membrane element rather than a fixed physical object. Physically, it can be understood as a local membrane area characterized by structural descriptors such as area per lipid, membrane thickness, hydration state, lipid composition, and mechanical properties. Depending on the experimental or computational framework, a pixel may correspond to a nanoscale membrane domain, a spatial region resolved by scattering or imaging measurements, or a computational discretization used to represent local electromechanical variables. The pixel concept therefore provides a multiscale framework for linking local structural descriptors to electrical work, energy storage and dissipation, thereby connecting membrane structure to adaptive electromechanical function. Such local structural variations, including differences in area per lipid, local curvature, hydration state, and compositional fluctuations, give rise to spatial heterogeneity that can influence both mechanical response and electrical behavior [36]. These structural variations evolve across a broad hierarchy of temporal scales. Local fluctuations in membrane thickness, dipolar orientation, and hydration may occur on nanosecond-to-microsecond timescales, whereas collective membrane undulations, domain rearrangements, and inter-bilayer structural relaxation can extend to milliseconds or longer. At still longer timescales, changes in lipid composition, protein distribution, metabolic state, and gene expression can remodel the architecture of myelin over hours, days, or even longer periods. Consequently, the structural organization of myelin should be viewed not as static, but as a continuously evolving landscape shaped by both rapid electrical activity and slower biological adaptation. In this sense, structure is not merely a scaffold but an active participant in determining how membranes respond to external stimuli.

In multilamellar assemblies such as myelin, structural organization also governs interlayer coupling and energy transfer pathways. The periodic stacking of bilayers creates a quasi-one-dimensional lattice in the out-of-plane direction, enabling coherent interactions between adjacent layers. These interactions are sensitive to hydration, as interfacial water layers mediate both repulsive and attractive forces between bilayers. Small changes in hydration can therefore lead to significant structural rearrangements, including swelling, compression, or transitions to non-lamellar phases [37].

Recent experimental observations of myelin swelling under conditions of damage provide direct evidence of the dynamic nature of this structural organization. Swelling reflects an increase in interlayer spacing driven by disruptions in ionic and fluid homeostasis, and its reversibility highlights the capacity of the multilamellar structure to reorganize and recover. Such structural remodeling is expected to alter the hydration state, membrane compressibility, dielectric response, and viscoelastic dissipation of the multilamellar membrane system, thereby influencing its electromechanical response. This behavior underscores the importance of treating structure as a dynamic variable coupled to both functional response and environmental conditions.

Experimentally, these structural adaptations can be quantified by measuring changes in lamellar repeat distance, membrane thickness, hydration profiles, interfacial roughness, domain organization, and correlated membrane undulation modes using neutron and X-ray scattering techniques. Correlating the evolution of these structural observables with electrical stimulation provides a direct experimental framework for testing how interlayer coupling and hydration-dependent structural remodeling influence electrical work, energy storage and dissipation, and adaptive electromechanical function in multilamellar membrane systems.

Importantly, structural parameters such as membrane thickness and area per lipid are not independent of function. Changes in these quantities alter the dielectric properties of the membrane, influence dipolar alignment, and modulate electromechanical coupling. In this context, the multilamellar architecture of myelin can be viewed as a spatially extended system in which local structural variations give rise to distributed functional responses.

Within the function-first multimodal framework proposed here, structural information is not treated in isolation but is integrated with functional and dynamical measurements. Structural descriptors, including membrane thickness, area per lipid, hydration state, interlamellar spacing, interfacial roughness, and lipid composition, serve as model inputs that are analyzed together with electrophysiological, mechanical, and spectroscopic measurements to infer latent electromechanical states governing membrane behavior. These inferred states are linked to experimentally measurable functional observables, including electrical response (e.g., capacitance hysteresis, conductance dynamics, and electrical work), viscoelastic dissipation, swelling and recovery kinetics, and spectroscopic signatures obtained from terahertz time-domain spectroscopy (THz-TDS), Raman spectroscopy, and neutron and X-ray scattering. By integrating high-resolution structural characterization with complementary functional and dynamical measurements, this framework establishes quantitative relationships between nanoscale membrane organization, collective molecular dynamics, and macroscopic electromechanical behavior.

We therefore view structure as a dynamic, spatially distributed field that encodes the state of the membrane and mediates its response to external stimuli. In multilamellar systems such as myelin, this field is shaped by hydration, composition, and interlayer interactions, and it evolves in time under the influence of electrical, mechanical, and thermal driving. Understanding this structural landscape is essential for linking microscopic organization to emergent phenomena such as energy buffering, memory, and adaptive behavior.

## 4. Dynamics: Collective Modes and Energy Flow

While structure defines the spatial organization of biological membranes, it is the dynamics of these systems that govern how energy is transported, stored, and transformed [38]. In multilamellar assemblies such as myelin, dynamics emerge from collective excitations that couple molecular degrees of freedom across length and time scales. These excitations provide the fundamental link between microscopic interactions and macroscopic functional behavior [39].

Collective vibrational modes, which can be broadly categorized into acoustic and optical phonons, play a central role in membrane dynamics [40]. In multilamellar membrane systems such as myelin, these collective excitations arise from coordinated motions of lipid molecules, lipid headgroups, hydrocarbon chains, and associated hydration layers, with coupling between adjacent bilayers extending these dynamics across the multilamellar structure. Acoustic modes correspond to long-wavelength, in-phase motions of neighboring lipid molecules or molecular groups and are associated with collective compression, bending, membrane undulations, and thickness fluctuations. These modes facilitate the propagation of mechanical and energetic disturbances across extended spatial domains and typically dominate the low-energy, low-frequency portion of the vibrational spectrum [41]. In contrast, optical modes arise from out-of-phase motions of neighboring lipid molecules or molecular substructures, particularly lipid headgroups, dipolar groups, hydrocarbon-chain segments, and interfacial hydration molecules. These modes generally occur at shorter wavelengths and higher frequencies and energies than acoustic modes and are strongly influenced by electrostatic interactions, hydration, lipid packing, and inter-bilayer coupling. Together, these collective excitations occupy the GHz–THz frequency range, corresponding approximately to sub-meV to tens-of-meV energies, and can be probed by inelastic X-ray scattering to obtain quantitative information on membrane elasticity, hydration-dependent interactions, molecular-scale dynamics, and energy transport in multilamellar membrane systems.

In multilamellar systems, interlayer coupling introduces additional complexity by enabling coherent interactions between adjacent bilayers. The resulting dynamical landscape can be viewed as a coupled lattice of oscillators, where energy is exchanged both laterally within individual bilayers and vertically across the multilamellar stack. In this context, optical modes may play a particularly important role in transient energy storage. Unlike acoustic modes, which primarily transport energy through collective deformations, optical modes can transiently localize energy within specific molecular and interfacial degrees of freedom, including lipid headgroups, dipolar networks, hydrocarbon chains, and hydration layers. Following electrical or mechanical stimulation, energy is initially coupled into these collective modes through membrane polarization, elastic deformation, and dipolar interactions. The lifetime of these excitations is governed by interlayer coupling, hydration-mediated interactions, and anharmonic coupling between vibrational modes, which together determine the rate at which energy is redistributed into lower-frequency collective modes and, ultimately, thermal degrees of freedom. Within the proposed framework, this transient population of optical modes represents a non-equilibrium redistribution of energy rather than simple heat accumulation or immediate vibrational relaxation. The resulting delayed relaxation provides a physical mechanism through which multilamellar membrane systems can buffer transient energetic perturbations and exhibit history-dependent electromechanical responses.

This process of energy storage and relaxation is inherently dissipative and occurs across a wide range of timescales [41]. Fast processes, on the order of picoseconds to nanoseconds, are associated with local molecular motions, dipolar relaxation, and hydration fluctuations, which manifest as spectroscopic signatures of collective molecular dynamics. Intermediate timescales involve collective membrane motions, membrane undulations, interlayer coupling, and structural relaxation processes, leading to measurable changes in membrane elasticity, correlated membrane fluctuations, and viscoelastic response. Slower processes, extending to milliseconds and beyond, reflect viscoelastic relaxation, changes in interlayer spacing, domain evolution, and larger-scale structural reorganization, which can be quantified through X-ray scattering measurements and mechanical characterization. The coexistence of these coupled dynamical processes gives rise to non-Markovian behavior, in which delayed relaxation across multiple timescales enables the system to retain a history of prior perturbations.

Myelin provides a particularly attractive system for exploring these ideas because its multilamellar architecture naturally supports coupling between adjacent bilayers across many repeating units. Rather than behaving as isolated membranes, the stacked layers of myelin form an extended dynamical network capable of supporting collective excitations over multiple length scales. The large number of coupled interfaces and hydration layers may therefore enhance both energy storage capacity and the lifetime of collective modes, making myelin especially well suited for buffering transient energetic perturbations [42].

**Figure 2:**
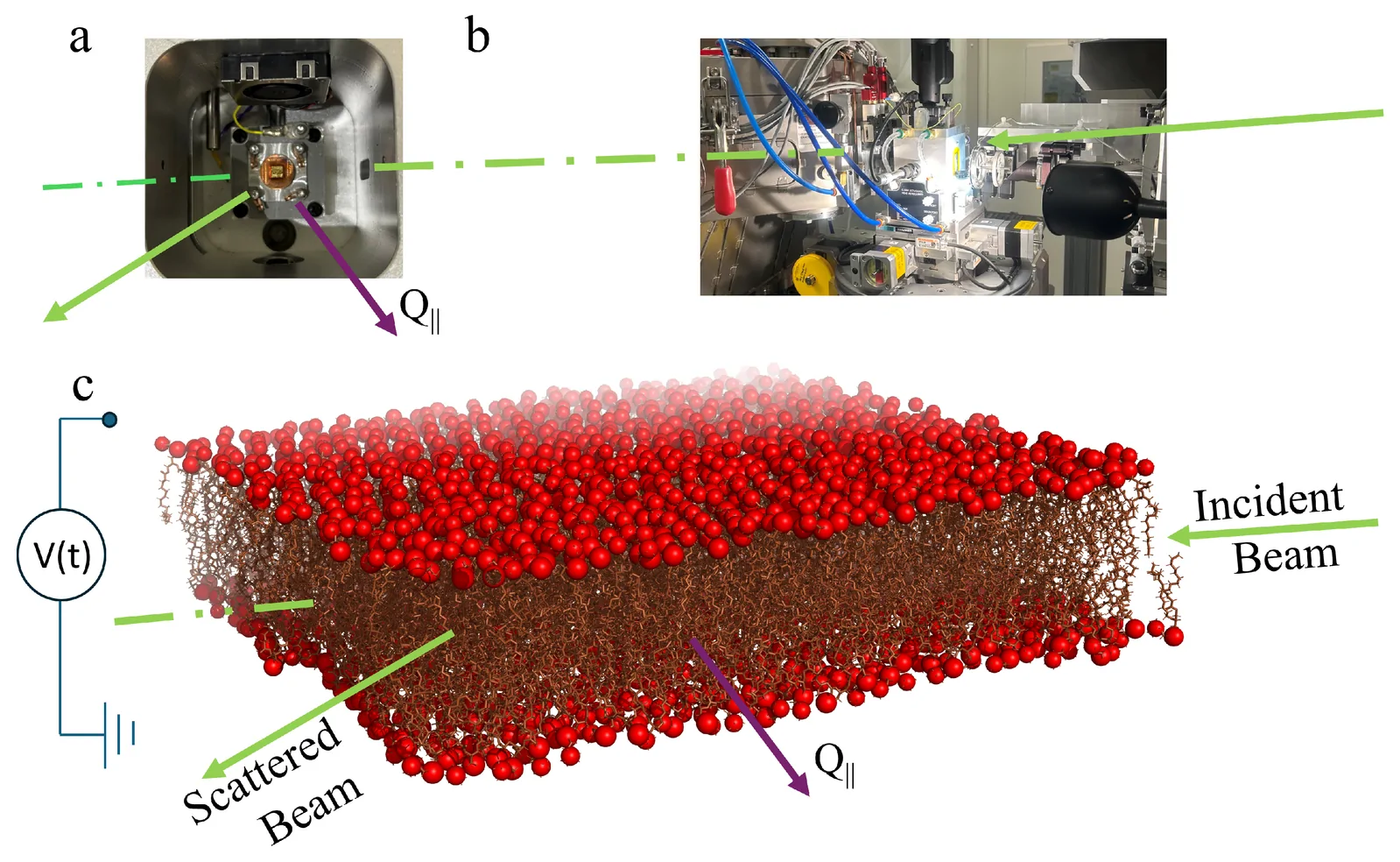
Original operando inelastic X-ray scattering (IXS) platform developed for investigating electrically stimulated multilamellar lipid membrane systems at the 10-ID beamline of NSLS-II, Brookhaven National Laboratory. (**a**) Top view of the electrode assembly supporting a multilamellar lipid stack during electrical stimulation. (**b**) Experimental IXS scattering geometry showing the incident and scattered X-ray beams used to perform momentum- and energy-resolved measurements under operando conditions. (**c**) Schematic of a multilamellar lipid membrane deposited on conductive electrodes and subjected to a time-dependent electrical bias, V(t) . The experimental configuration enables simultaneous electrical stimulation and IXS measurements, providing access to the dynamic structure factor, phonon dispersion relations, and collective vibrational excitations while probing both in-plane ( $Q_{\|}$ ) and out-of-plane membrane dynamics. This platform enables direct investigation of electromechanical coupling, collective energy transport, and non-equilibrium dynamics in biologically relevant multilamellar membrane systems.

Experimental access to these dynamic processes is enabled by a combination of spectroscopic and scattering techniques. Figure 2 presents our original operando inelastic X-ray scattering (IXS) platform developed for investigating electrically stimulated multilamellar membrane systems under controlled experimental conditions. The platform consists of a multilamellar lipid sample positioned between conductive electrodes, enabling simultaneous electrical stimulation and momentum- and energy-resolved IXS measurements. The primary experimental observable is the dynamic structure factor, from which phonon dispersion relations and collective vibrational excitations are determined under operando conditions. By resolving both acoustic and optical branches, IXS enables quantitative characterization of collective excitations and reveals how membrane composition, hydration state, interlayer coupling, and external electrical stimulation influence energy transport and redistribution within multilamellar membrane systems. Terahertz time-domain spectroscopy (THz-TDS) provides complementary information by probing low-energy collective motions and dipolar fluctuations, offering additional insight into the coupling between hydration dynamics and membrane vibrations. Together, these techniques provide a multiscale characterization of membrane dynamics, bridging molecular motions with macroscopic electromechanical response [26].

A central hypothesis emerging from this framework is that biological membranes do not simply dissipate energy but actively redistribute and transiently retain it through collective excitations. Within this hypothesis, the relaxation of optical modes into structural degrees of freedom, including membrane undulations, dipolar domains, and hydration-mediated configurations, may provide a mechanism for energy buffering across multiple timescales. Because these relaxation pathways are delayed and history-dependent, the subsequent response of the system depends on its previous energetic state. In this view, membrane memory emerges not from a single molecular component but from the collective dynamics of the membrane itself.

We emphasize that this proposed relationship between collective vibrational dynamics, transient energy buffering, and membrane memory represents a testable hypothesis rather than an established mechanism in myelin. If correct, the framework predicts several experimentally observable signatures, including stimulation-dependent changes in phonon dispersion relations, altered collective mode lifetimes, hysteretic spectral shifts, delayed relaxation following removal of the electrical stimulus, and history-dependent evolution of the dynamic structure factor. Observation of these signatures using operando inelastic X-ray scattering and complementary spectroscopic techniques would provide direct experimental tests of the proposed electromechanical memory framework.

Importantly, these processes are strongly influenced by hydration and temperature, highlighting the central role of thermal energy in coupling electrical, mechanical, structural, and dynamical degrees of freedom. Thermal fluctuations continuously drive molecular motion, hydration dynamics, membrane undulations, and collective excitations, thereby influencing how energy is redistributed throughout the system. In this sense, membrane dynamics can be viewed as a continuous exchange of energy among different modes and degrees of freedom, governed by both intrinsic material properties and external perturbations.

Within the proposed framework, electrical or mechanical stimulation first perturbs membrane polarization and excites collective vibrational modes. These primary dynamical responses are subsequently coupled to structural degrees of freedom, including hydration, membrane undulations, interlayer coupling, and lipid organization, which relax over different timescales. The resulting delayed structural and dynamical relaxation gives rise to history-dependent behavior that can be quantified through hysteresis, electrical work, structural relaxation, and stimulation-dependent changes in collective vibrational spectra.

Within the function-first multimodal framework proposed here, these structural, dynamical, and functional observables are integrated to construct a comprehensive description of membrane behavior. High-dimensional datasets capturing temporal evolution, frequency response, and environmental dependence can be analyzed using data-driven approaches to identify correlations and infer the underlying mechanisms linking energy input, structural adaptation, and functional response. This integration enables the mapping of dynamic states onto functional outcomes, providing a pathway to predict and ultimately control membrane behavior under non-equilibrium conditions.

We therefore interpret multilamellar membrane systems as dynamically adaptive networks in which collective modes mediate the flow and storage of energy. In the myelin sheath, this dynamic landscape underpins not only efficient signal propagation but also resilience to perturbation and the capacity for structural remodeling. By linking collective excitations to energy buffering and memory, this framework provides a unified physical basis for understanding how biological membranes operate far from equilibrium.

## 5. Unified Framework: From Energy to Memory

The preceding sections establish function, structure, and dynamics as interconnected but often independently studied descriptors of membrane behavior. Here, we synthesize these elements into a unified physical framework in which biological membranes, and in particular multilamellar systems such as myelin, are understood as adaptive, energy-processing media capable of encoding and retaining information.

At the core of this framework lies the principle that energy flow governs state evolution. In biological systems, multilamellar membranes such as myelin do not exist in isolation but form part of a larger electromechanical and metabolic network that includes axons, glial cells, and the surrounding extracellular environment. External stimuli, including electrical activity associated with action-potential propagation, mechanical perturbations, metabolic fluctuations, and changes in hydration, continuously inject energy into the membrane system [43,44]. This energy is not instantaneously dissipated but is redistributed across multiple degrees of freedom, including dipolar alignment, membrane deformation [44], hydration dynamics, and collective vibrational modes [41]. The interplay between these processes defines the system’s trajectory through its configurational space [45].

Structure provides the spatial constraints within which this energy redistribution occurs. Variations in membrane thickness, area per lipid, hydration state, and interlayer spacing define a heterogeneous landscape that modulates local energy storage and transfer. In multilamellar systems, inter-bilayer coupling extends this landscape into the out-of-plane direction, enabling energy exchange across layers and creating a quasi-lattice of interacting units. This spatial organization gives rise to a distributed network in which local structural variations can influence global behavior [46].

As one illustrative example, hydration-induced swelling or electrically driven structural remodeling of a multilamellar membrane may transiently increase the interlayer spacing, thereby modifying inter-bilayer coupling, dielectric properties, and membrane viscoelasticity. Following removal of the perturbation, structural relaxation may proceed over finite timescales, resulting in delayed recovery of the interlayer spacing and associated dielectric, viscoelastic, and scattering observables. Within the proposed framework, this sequence illustrates how structural evolution through the membrane energy landscape can generate measurable history-dependent responses.

More generally, the dynamics of collective excitations determine how energy moves through this network. Acoustic modes propagate energy over long distances, while optical modes act as localized reservoirs that temporarily store energy before releasing it into lower-energy configurations [41]. The relaxation of these modes is inherently time-dependent and often slow compared to the timescale of external driving, leading to a mismatch between input and response. This mismatch manifests as hysteresis, delayed relaxation, and path-dependent behavior.

Function emerges as the observable consequence of these coupled processes [12]. Measurements such as current–voltage hysteresis, charge–voltage loops, viscoelastic dissipation, and spectroscopic signatures capture the system’s response to external stimuli, reflecting both its instantaneous state and its history. In this context, functional observables serve as experimentally accessible projections of the underlying structural and dynamical state.

Within the proposed framework, delayed relaxation alone is not sufficient to establish memory-like behavior, as similar relaxation processes are common in soft condensed matter systems. Instead, memory-like behavior is operationally defined by history-dependent responses in which the system’s behavior depends on its prior stimulation history rather than solely on its instantaneous state. Experimentally, such behavior may be identified through path-dependent responses, reproducible hysteresis over repeated stimulation cycles, delayed recovery following removal of the stimulus, or altered responses to subsequent perturbations. Together, these criteria provide a minimum experimental definition of adaptive memory-like behavior, distinguishing it from ordinary relaxation dynamics and establishing experimentally testable benchmarks for evaluating the proposed framework.

We therefore interpret memory in biological membranes as a natural consequence of energy storage and delayed relaxation. When energy is temporarily stored in collective modes or structural configurations, the system retains information about past stimuli. This information is encoded in the spatial distribution of dipoles, the configuration of hydration layers, the arrangement of membrane domains, and the collective state of coupled bilayers. As the system relaxes, this stored energy is gradually released, influencing future responses and giving rise to non-Markovian dynamics [19].

A key aspect of this framework is the interplay among different forms of energy that govern membrane dynamics. Passive thermal fluctuations continuously drive equilibrium molecular motion, hydration dynamics, membrane undulations, and collective excitations, thereby facilitating energy redistribution throughout the system [41,47]. In contrast, externally applied electrical or mechanical stimuli perform work on the membrane, driving it away from equilibrium and initiating structural and dynamical adaptation. During subsequent relaxation, part of this externally supplied energy is dissipated as heat, while another portion may remain transiently stored in collective modes or structural configurations. In living systems, these processes occur within a metabolically maintained non-equilibrium environment, where active cellular processes continuously supply energy to sustain ionic gradients, membrane potentials, and physiological function. Thus, membrane behavior reflects the interplay among thermal fluctuations, externally applied work, dissipative processes, and metabolic activity, governed by both intrinsic material properties and external stimuli. This interplay underlies the adaptive and emergent properties of biological membranes.

### Data-Driven Integration of Structure, Dynamics, and Function

A central challenge in membrane biophysics is that structure, dynamics, and function are often measured independently despite being strongly coupled. Structural measurements provide information about membrane organization, hydration, and multilamellar architecture. Dynamical measurements reveal collective excitations, relaxation pathways, and energy transport mechanisms. Functional measurements capture the observable response of the system to external stimuli. Integrating these complementary observables into a unified description requires approaches capable of handling complex datasets spanning multiple length and time scales.

Within the framework proposed here, functional measurements serve as the primary observables, while structural and dynamical measurements provide mechanistic constraints on the underlying state of the system. By combining electrophysiology, neutron and X-ray scattering, terahertz spectroscopy, and computational analysis, it becomes possible to reconstruct effective energy landscapes governing membrane behavior. Such integrated approaches provide a pathway toward identifying latent variables, mapping energy flow pathways, and predicting how structural adaptation and collective dynamics influence functional response.

**Figure 3:**
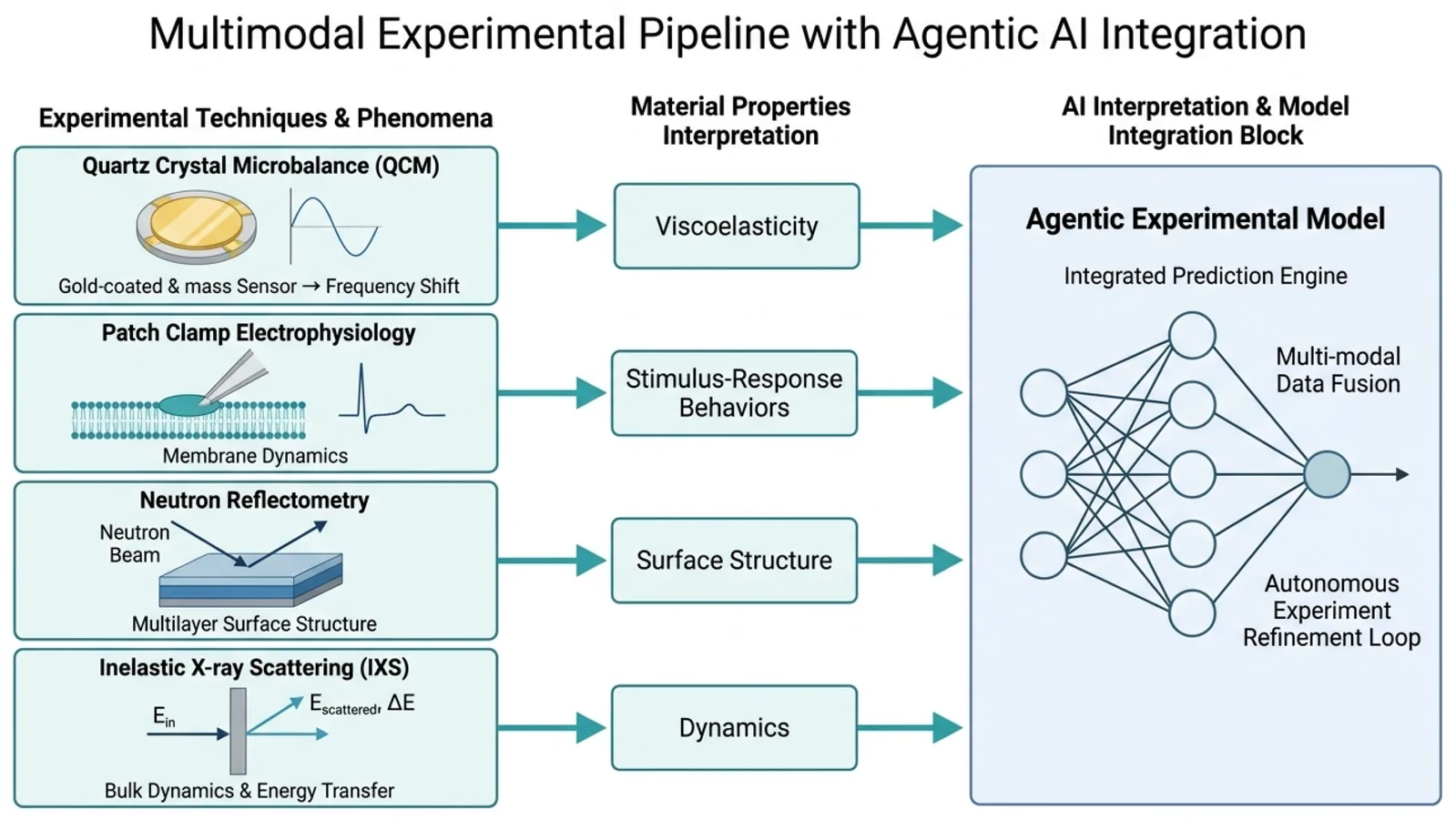
Multimodal experimental pipeline for integrating structure, dynamics, and function through data-driven modeling. Functional measurements, including electrophysiology and quartz crystal microbalance with dissipation (QCM-D), provide experimentally measurable observables related to ionic transport, electrical response, hydration-mediated adaptation, and viscoelasticity. Structural measurements obtained by neutron reflectometry characterize membrane organization, interlayer spacing, hydration profiles, membrane thickness, and nanoscale heterogeneity, while inelastic X-ray scattering probes collective dynamics, phonon-mediated energy transport, and vibrational excitations within multilamellar membrane systems. These complementary measurements provide inputs for computational integration, where machine intelligence and data-driven modeling infer latent variables describing the underlying membrane state, including structural organization, collective dynamics, energy storage, and relaxation pathways. The inferred physical state is subsequently used to reconstruct effective energy landscapes and predict experimentally measurable biological and material responses, including hysteresis, metastable states, delayed relaxation, energy buffering, and adaptive memory-like behavior. Together, this workflow provides a unified framework for understanding biological membranes as non-equilibrium, energy-processing systems in which structure, dynamics, and function are intrinsically coupled across multiple spatial and temporal scales.

The proposed workflow begins with electrophysiological measurements (Figure 3), which provide functional inputs, including current–voltage hysteresis, charge–voltage loops, and relaxation kinetics. Neutron and X-ray scattering characterize membrane structure, hydration, interlayer organization, and nanoscale architecture, while terahertz spectroscopy and inelastic X-ray scattering probe collective vibrational dynamics, phonon dispersion, and energy transport. Computational analysis integrates these complementary datasets to infer latent variables describing the membrane state, including structural organization, collective excitations, energy storage, and relaxation pathways. The resulting models are validated by predicting experimentally measurable observables across modalities, such as hysteresis evolution, relaxation dynamics, spectroscopic signatures, and scattering responses obtained under independent stimulation protocols.

This integrated approach transforms the study of biological membranes from a descriptive to a predictive science. By iteratively refining models based on experimental observations, it becomes possible to identify the pathways through which energy flows, is stored, and is released, and to relate these pathways directly to functional outcomes. In doing so, we move toward a quantitative understanding of how memory and adaptation emerge in soft, non-equilibrium systems.

We therefore propose that multilamellar biological membranes should be viewed as adaptive energy-processing systems in which structure defines the accessible states, dynamics govern transitions between those states, and function provides an observable record of the system’s history. In the case of myelin, this perspective provides a new framework for understanding how energy storage, delayed relaxation, and structural adaptation contribute to both physiological function and the early stages of disease. Because myelin is intimately coupled to electrically active axons, this framework further suggests that action-potential-driven energy flow may influence not only signal propagation but also the long-term structural and dynamical state of the sheath itself.

By unifying structure, dynamics, and function within a single framework, this perspective establishes a foundation for exploring biological membranes as adaptive, information-processing systems and for designing bio-inspired materials that harness similar principles.

## 6. Outlook: From Biological Membranes to Adaptive Materials and Disease

The framework developed here positions biological membranes, and in particular multilamellar systems such as myelin, as adaptive, energy-processing media in which structure, dynamics, and function are intrinsically coupled. This perspective opens several avenues for future research spanning fundamental biophysics, neuroscience, and the design of bio-inspired materials.

One immediate implication concerns the understanding of neurological disorders associated with myelin dysfunction, such as multiple sclerosis (MS) and other demyelinating conditions. Traditional descriptions of these diseases focus on structural degradation and immune-mediated damage. In addition to structural degradation, experimental and clinical studies have reported dynamic myelin swelling, activity-dependent instability, and partial structural recovery, indicating that myelin remodeling occurs during the early stages of disease progression. These observations suggest that at least some aspects of early myelin dysfunction remain reversible before extensive structural deterioration develops.

Within the framework proposed here, these observations may be interpreted as manifestations of altered energy flow, hydration balance, electromechanical coupling, and collective membrane dynamics. In particular, myelin swelling may represent an early non-equilibrium response associated with transient changes in hydration, interlayer organization, and energy redistribution, rather than solely a marker of structural deterioration. From this perspective, partial structural recovery may reflect the restoration of membrane homeostasis as energy is redistributed and dissipated across coupled structural and dynamical degrees of freedom.

If validated experimentally, this framework may suggest new therapeutic opportunities aimed at stabilizing membrane function before irreversible structural damage occurs. The sensitivity of myelin to neuronal activity further highlights the importance of coupling between electrical signaling and membrane dynamics. Action-potential-driven ionic fluxes may transiently redistribute ions and water across the periaxonal space, leading to changes in hydration, lamellar spacing, membrane tension, dielectric properties, and the mechanical state of the multilamellar sheath. These coupled physical changes may, in turn, influence collective membrane dynamics, electromechanical coupling, and activity-dependent regulation by oligodendrocytes, thereby affecting the structural stability of myelin. Increased neuronal activity can exacerbate these processes, while reduced activity may mitigate them, suggesting that electrical signaling and membrane structure are intimately linked. Rather than directly manipulating neuronal firing patterns, future therapeutic approaches may focus on the myelin sheath itself by targeting oligodendrocyte physiology, ion regulation, hydration balance, and electromechanical coupling. Such interventions could help stabilize multilamellar organization, restore energy buffering capacity, and promote structural recovery before irreversible damage occurs.

A particularly promising direction involves operando characterization of membrane systems under controlled electrical, mechanical, and chemical stimulation. By combining electrophysiology with neutron reflectometry, inelastic X-ray scattering, terahertz spectroscopy, and complementary structural probes, it becomes possible to observe how membranes evolve in real time while driven away from equilibrium. As one representative experimental design, electrically stimulated myelin-mimetic multilamellar lipid stacks could be monitored simultaneously for changes in lamellar spacing, viscoelastic relaxation, electrophysiological response (e.g., current–voltage hysteresis), and collective vibrational dynamics under well-defined stimulation protocols. Correlating these complementary observables would enable direct testing of how electrical stimulation redistributes energy across structural, mechanical, and dynamical degrees of freedom, and whether these changes give rise to history-dependent membrane adaptation. Such experiments provide a direct route toward linking structure, dynamics, and function within a single experimental framework and may enable the identification of early biophysical signatures of disease before substantial structural loss has occurred.

Beyond disease, the principles outlined in this work provide a foundation for the development of bio-inspired adaptive materials. Multilamellar lipid systems exhibit properties that are highly desirable for next-generation technologies, including energy buffering, nonlinear response, and memory-like behavior. More broadly, the concepts developed here suggest a new class of adaptive materials capable of storing, redistributing, and processing energy through collective dynamics. Unlike conventional materials designed around static properties, such systems would continuously evolve in response to external stimuli and retain information about prior states. By exploiting electromechanical coupling, hydration-mediated interactions, and collective excitations, it may become possible to engineer materials that exhibit memory, adaptability, and self-organizing behavior, enabling applications in neuromorphic computing, sensing, and energy-efficient information processing.

The integration of multimodal experimental techniques and data-driven analysis represents a critical step toward advancing both biological and materials applications. The combination of functional measurements, including electrophysiology and QCM-D, structural characterization through neutron and X-ray scattering, and dynamical probes such as IXS and THz spectroscopy generates rich, high-dimensional datasets that capture the full complexity of membrane behavior. Integrating these complementary observables within automated experimental and computational workflows enables exploration of parameter spaces that would otherwise be inaccessible and provides a pathway toward predictive descriptions of non-equilibrium membrane systems.

Looking forward, a key challenge will be to establish quantitative links between microscopic variables and macroscopic observables. Within the proposed framework, priority microscopic variables include lipid composition, hydration state, interlayer spacing, and collective vibrational dynamics (phonon spectra), while key macroscopic observables include electrical response (e.g., current–voltage hysteresis and membrane capacitance), mechanical stability, and functional performance. These variables are closely interconnected: lipid composition and hydration govern membrane packing and interlayer spacing, which influence membrane mechanics, collective vibrational dynamics, and ultimately the electrical response. Establishing quantitative relationships among these interconnected variables will require coordinated efforts across disciplines, combining advances in experimental techniques, theoretical modeling, and computational analysis. Success in this direction would provide a unified description of how energy flows through biological membranes, how it is temporarily stored, and how it influences future behavior.

In the near term, the most immediate opportunities lie in experimentally validating the proposed framework through coordinated multimodal measurements of electrically stimulated membrane systems. Establishing quantitative relationships among membrane structure, collective dynamics, electrical response, and mechanical properties will provide a critical foundation for testing the underlying physical hypotheses and developing predictive models of membrane adaptation. These experimentally accessible objectives represent an important first step toward understanding non-equilibrium membrane behavior.

Beyond these near-term objectives, biological membranes may be viewed as prototypes of adaptive matter: systems that continuously exchange energy with their environment, reorganize their internal structure, and retain information about previous states. The myelin sheath represents a particularly compelling example because it combines a highly ordered multilamellar architecture with dynamic remodeling, electromechanical responsiveness, and long-lived collective behavior. Understanding these properties may provide new insights into both biological function and, ultimately, the design of next-generation adaptive materials.

We anticipate that the continued integration of functional measurements, multimodal structural characterization, and dynamical probes will enable the development of predictive frameworks capable of linking microscopic organization to emergent behavior. As an initial experimental priority, future studies should focus on a core set of measurable variables, including hydration state, interlayer spacing, lipid and protein composition, capacitance hysteresis, collective mode lifetimes, and recovery kinetics. Together, these complementary observables capture structural, electrical, mechanical, and dynamical aspects of membrane behavior, providing a practical foundation for identifying latent variables and reconstructing the energy landscape underlying non-equilibrium membrane adaptation. Such approaches may ultimately reveal how biological membranes store, redistribute, and utilize energy to adapt to changing environments.

We emphasize that the proposed interpretation of myelin swelling, recovery, and disease as manifestations of altered energy redistribution remains a working hypothesis that requires direct experimental validation. A key priority for future studies will be to simultaneously monitor lamellar spacing, hydration state, viscoelastic relaxation, and electrophysiological response during controlled swelling and recovery using complementary operando measurements. Establishing quantitative relationships among these observables will provide a critical test of the proposed framework and help determine whether changes in energy redistribution are associated with membrane adaptation during normal physiological function and disease.

## 7. Discussion and Conclusions

In this article, we have advanced a unified framework for understanding biological membranes as adaptive, energy-processing systems in which structure, dynamics, and function are intrinsically coupled. Building upon the established view of myelin as a dynamic, activity-responsive biological structure, our framework provides a unified physical description that links electrical stimulation, membrane mechanics, hydration, collective vibrational dynamics, and structural organization through energy flow, while establishing experimentally testable relationships between microscopic membrane properties and macroscopic functional behavior. We propose that multilamellar assemblies, such as the myelin sheath, operate far from equilibrium and that the coupling among these processes gives rise to emergent behaviors, including energy buffering, nonlinear response, and memory-like adaptation.

A central element of this framework is the function-first paradigm, in which observable responses serve as the primary entry point for probing membrane behavior. These responses reveal complex, history-dependent phenomena that cannot be fully explained within equilibrium descriptions. Instead, they reflect the interplay between electrical stimulation, mechanical deformation, hydration dynamics, collective excitations, and structural adaptation across multiple spatial and temporal scales.

By integrating structural organization with collective dynamics, we establish a multiscale picture in which membranes are described as spatially heterogeneous, dynamically evolving systems. Within this picture, multilamellar architectures introduce interlayer coupling and extend the system into a quasi-lattice, enabling coherent interactions and distributed energy transfer. Hydration emerges as a key control parameter, modulating both structural organization and dynamical response while coupling molecular processes to larger-scale collective behavior.

The concept of memory arises naturally within this framework as a consequence of energy storage and delayed relaxation. Energy injected into the system is redistributed across collective modes, including acoustic and optical excitations, and temporarily stored in structural and dipolar configurations. The subsequent relaxation of these modes occurs over extended timescales, leading to hysteresis, path dependence, and non-Markovian behavior. In this sense, memory is not an additional feature imposed on the system, but an intrinsic property of its dynamic evolution.

Importantly, this perspective provides a new lens through which to interpret biological function and dysfunction. In the case of myelin, structural remodeling, swelling, and recovery can be understood as manifestations of shifts in energy distribution and coupling between electrical activity, ion homeostasis, hydration, and collective dynamics. Disruptions to these processes may precede substantial structural deterioration, suggesting that early stages of disease are governed by dynamic instability and altered energy flow. This insight raises the possibility that restoring homeostasis, stabilizing collective dynamics, and preserving energy buffering capacity may provide new avenues for therapeutic intervention.

The framework developed here also highlights the importance of integrating functional, structural, and dynamical information within a common description of membrane behavior. Such integration provides a pathway toward identifying latent variables, reconstructing effective energy landscapes, and developing predictive models capable of linking microscopic organization to macroscopic response. More broadly, it enables a transition from descriptive studies of membrane systems toward quantitative frameworks capable of explaining adaptation, memory, and emergent behavior.

Looking ahead, several challenges and opportunities remain. Establishing quantitative relationships between microscopic variables and macroscopic observables will require advances in both experimental resolution and theoretical modeling. Extending these concepts to increasingly complex biological systems, including neurons, glial networks, and organoids, will be critical for validating their physiological relevance. At the same time, the principles outlined here provide a foundation for the design of bio-inspired materials that harness energy buffering, collective dynamics, and memory-like behavior for applications in sensing, computation, and adaptive systems.

In conclusion, we propose that biological membranes, and in particular multilamellar systems such as myelin, should be understood as non-equilibrium adaptive materials capable of storing, redistributing, and utilizing energy through collective dynamics. By unifying function, structure, and dynamics within a framework centered on energy flow, delayed relaxation, and memory, this Perspective provides a pathway toward a deeper understanding of biological organization and offers a blueprint for the development of next-generation adaptive and memory-enabled materials inspired by biology.

## Data Availability

The original contributions presented in the study are included in the article. Further inquiries can be directed to the corresponding authors.
